# H5Nx Panzootic Bird Flu—Influenza’s Newest Worldwide Evolutionary Tour

**DOI:** 10.3201/eid2302.161963

**Published:** 2017-02

**Authors:** Jeffery K. Taubenberger, David M. Morens

**Affiliations:** National Institutes of Health, Bethesda, Maryland, USA (J.K. Taubenberger, D.M. Morens);; Associate Editor, Emerging Infectious Diseases, Atlanta, Georgia, USA (D.M. Morens)

**Keywords:** influenza, influenza viruses, viruses, panzootic, enzootic, H5Nx subtype, reassortment, evolution

Influenza A viruses (IAVs) cause annual epidemics, periodic pandemics, and enzootic infections of numerous animals, including horses, dogs, pigs, seals, and whales (*1*). The natural reservoir for IAV is wild aquatic birds, including diverse species of Anseriformes (ducks and geese) and Charadriiformes (shorebirds and gulls), which continually transport an incredible array of genetically diverse IAVs over vast distances during migration.

In wild birds, IAVs usually cause inapparent, self-limited, lower gastrointestinal tract infections. Such low pathogenicity avian influenza (LPAI) viruses represent most of the avian influenza viruses. IAVs (mostly of low pathogenicity) also occasionally host switch to domestic poultry (mainly chickens and turkeys). Because gallinaceous poultry are not natural hosts of IAVs, sustained epizootic and enzootic transmission in poultry leads to viral genetic changes not found in IAVs adapted to other hosts, such as wild birds or mammals.

IAVs have 1 of 18 hemagglutinin (HA) subtypes (HA being the major surface glycoprotein that elicits immune responses). Two of these subtypes, H5 and H7, can spontaneously undergo mutations at the HA cleavage site to become highly pathogenic avian influenza (HPAI) viruses for gallinaceous poultry, typically resulting in fatal systemic infection in poultry and sometimes in wild birds that come into contact with poultry. We should emphasize that the designation of highly pathogenic and low pathogenicity refers only to bird infections and has no bearing on whether human exposure to such viruses can cause symptomatic or severe disease.

Although LPAI poultry epizootics may not be recognized because infections are asymptomatic, fatal HPAI outbreaks in domestic chicken and turkey flocks are obvious and have been described worldwide for >225 years. An Asian lineage of HPAI H5N1, designated A/goose/Guangdong/1/1996 H5N1, emerged in 1996 and has since spread throughout much of Asia, Europe, the Middle East, and Africa, causing almost 900 serious human infections and >400 deaths. This alarmingly high number probably does not represent virulence for or adaptation to humans but rather the existence of rare host susceptibilities to AIVs, considering that millions of persons have been exposed (*2*). In the past 2 decades, this H5N1 lineage has developed into multiple sublineages and has undergone multiple reassortment events leading to major alteration of internal genes but until recently has retained its original N1 subtype neuraminidase (NA).

During 2013–2015, a sublineage of HPAI H5N1, referred to as clade 2.3.4.4, which had first been reported in 2008 in China, suddenly spread explosively to birds in much of the rest of the world (*3*,*4*). In doing so, this sublineage underwent genetic reassortment with various naturally occurring LPAI viruses and repeatedly switched out its long-stable N1 subtype for importations of several new NA subtypes, including N2, N3, N5, N6, and N8. This unprecedented series of events resulted in multiple so-called H5Nx viruses (i.e., H5 clade 2.3.4.4 viruses coupled with any NA subtype that reassorted into the preexisting complex of viral genes). During 2013–2015, H5Nx viruses spread panzootically outward from China (*5*). Exported H5N6 viruses predominated in Asia, and H5N8 viruses were exported in independent sublineages westward to Europe and eastward to North America. In North America, H5N8 virus reassorted into H5N1 and H5N2 viruses, spreading in early 2015 to 21 states in the United States and causing the loss of >50 million poultry, at a cost of $5 billion. After ≈6 months, these viruses all but disappeared from North America and receded dramatically in Europe. In southern China, however, H5N6 has become so widespread in duck populations that it has largely replaced H5N1 as the dominant AIV seen in poultry markets (*4*).

During summer 2016, H5N8 clade 2.3.4.4 viruses once again began spreading explosively in a second panzootic wave along migratory bird pathways from Russia/Mongolia, separately into Europe and North Africa, the Middle East, and India (*6*). At the same time, H5N6 clade 2.3.4.4 viruses continue spreading throughout Asia, and there are fears that North America will experience similar resurgent panzootic waves. In the original 2014–2015 panzootic wave, wild bird deaths were uncommon, as is expected for poultry-adapted HPAI viruses. In contrast, the still-ongoing 2016 European/African/Middle Eastern/Indian resurgent panzootic wave has resulted in the deaths of many ducks and a wide variety of wild bird species. 

Historically, HPAI viruses are believed to spread by high-production poultry farming and the movements of infected birds, bird material, or surface contamination from infected flocks to susceptible flocks. However, the aggressive spread of the new HPAI H5Nx viruses in migratory birds along established flyways after breeding and molting seasons strongly implicates migratory wild birds in the 2 recent panzootic waves. Although genetic bases for panzootic explosivity have not been demonstrated, scientists speculate that H5Nx viruses have become more transmissible than other IAVs or more stable in the environment or in wild birds over long migrations.

In this context, the study of Guo et al. (*7*) in this issue of Emerging Infectious Diseases is of particular interest. It has long been believed that changes in the HA receptor-binding domain are key to viral adaptation in new hosts, specifically enabling wild bird–origin IAVs to adapt to domestic poultry, to mammals, and to humans. Avian-adapted IAVs usually bind preferentially to glycan receptors that terminate in a sialic acid bound to an underlying galactose with an α2,3 linkage. NA acts to cleave cell-bound and virion-bound sialosides, enabling newly formed virions to be released. Because HA binds sialosides while NA cleaves them, it has long been assumed that a functional balance is required between HA and NA activity (*4*).

Glycan array studies have demonstrated that modifications to the antepenultimate sugar in these sialosides (e.g., sulfation or fucosylation) often affect HA binding affinity. HA binding to α2,3-fucosylated sialosides, which may be a feature of galliform poultry adaptation (*8*) should be examined more widely in both wild bird and in poultry-adapted IAVs of a variety of HA subtypes.

The study by Guo et al. (*7*) looked specifically at H5 clade 2.3.4.4 mutations and sialic acid receptor binding properties associated with emergence and spread of a 2014 European chicken H5N8 virus. The authors report mutations in HA residues 222 and 227 (H3 subtype numbering) associated with a change in HA receptor binding specificity. In comparisons of HA glycan array binding properties of an ancestral H5N1 to those of the newer mutated H5N8 clade 2.3.4.4 virus, the newer HA retained its ability to bind to nonfucosylated sialosides while at the same time acquiring the ability to bind to several α2,3-fucosylated sialosides. Examination of additional H5Nx viruses from wild birds and domestic poultry is necessary to understand the prevalence of these binding patterns in H5 clade 2.3.4.4 H5Nx viruses.

The unexpected acquisition of this new property raises several questions. Is the sudden emergence and spread of H5Nx lineage viruses related directly to fucosylated sialoside binding, to the blended HA binding specificity observed, to H5 pairing with new NAs, or to other unappreciated genomic mutations acting alone or in concert? The founder A/goose/Guangdong/1/1996 H5N1 lineage, despite multiple reassortment events over a 20-year period, did not replace its N1 subtype until the recent emergence of H5Nx viruses. Did altered receptor specificity for fucosylated sialosides enable H5 to efficiently partner with a variety of different NA subtypes that ancestral H5 viruses were unable to incorporate into their gene complex via reassortment, because of some yet-unappreciated HA/NA functional mismatch? Are the H5Nx viruses more transmissible or more stable in wild bird species, or more environmentally stable? Is the HPAI phenotype expressed differently than it is with ancestral H5N1 lineages (e.g., in pathogenic effects on a different spectrum of wild bird species)?

What are the implications for humans, who are not commonly productively infected by LPAI or HPAI poultry viruses? Mammalian (and human) adaptation is associated with non-fucosylated sialosides especially with α2,6-linked sialoside receptor binding. If enhanced binding to fucosylated sialosides occurs in these poultry-adapted viruses without changing the overall binding preference for α2,3-linked sialoside (characteristic of galliform poultry adaptation), then these viruses are presumably less capable of back-adapting to pose a risk for humans. At the same time, lectin histochemical studies should be performed to look for the presence and distribution of fucosylated sialosides along the respiratory tract of humans, in mammals that either sustain IAV enzootic spread or that can be infected experimentally (e.g., swine, horses, ferrets) and in wild and domestic bird species.

How IAVs evolve, switch hosts, and stably adapt to new hosts remain poorly understood but undoubtedly reflects multiple independent pathways. A better understanding of the molecular bases of these host-adaptation events may help us to recognize genetic signatures of emerging IAVs that can infect humans, domestic animals, and wildlife and to better prevent and control transmission. 

In addition to providing insight on the mechanisms by which a novel panzootic virus is emerging, the study of Guo et al. (*7*) reminds us of the ability of the influenza virus to surprise us with a remarkable repertoire of multidirectional evolution, which presents us with newer and more complicated challenges. In the past several decades, influenza viruses have been moving about globally in new and different ways. If we hope to control them, we need to understand what they are doing, and how they are doing it.
